# Recurrent Concurrent Diabetic Ketoacidosis and Thyroid Storm

**DOI:** 10.7759/cureus.14273

**Published:** 2021-04-03

**Authors:** David F Crudo, Elizabeth T Walsh, Janel D Hunter

**Affiliations:** 1 Pediatric Endocrinology, Wake Forest University School of Medicine, Winston-Salem, USA

**Keywords:** diabetic ketoacidosis, thyroid storm, thyrotoxicosis, autoimmune thyroid disease

## Abstract

Diabetic ketoacidosis (DKA) and thyroid storm are serious complications of underlying disease states. Either condition can induce the other, and the co-occurrence of these conditions is uncommon. We present the case of an adolescent patient with type 1 diabetes and autoimmune hypothyroidism who developed recurrent concurrent DKA and thyroid storm twice in an eight-month period. The simultaneous development of DKA and thyroid storm is uncommon with only 28 cases previously reported. Co-presentation of these two life-threatening conditions occurs in people with either preexisting diabetes, thyroid disease, or both. The purported pathophysiology of how DKA and thyroid storm affect the other is discussed.

## Introduction

Diabetic ketoacidosis (DKA) is a serious life-threatening complication of diabetes mellitus in which severe insulin deficiency leads to hyperglycemia, metabolic acidosis, dehydration, and electrolyte imbalances. The initial onset of diabetes, omissions in insulin therapy, and infections are common triggering factors [1]. Thyroid storm is an acute complication of hyperthyroidism typically precipitated by uncontrolled hyperthyroidism, discontinuation of antithyroid drugs, surgery, infection, trauma, or metabolic derangements [2]. DKA can be induced by thyroid storm and vice versa [3]. Although the co-occurrence of these entities is uncommon, each is potentially life-threatening without prompt recognition and appropriate therapy.

## Case presentation

The patient is a 15-year-old female with an eight-year history of type 1 diabetes mellitus and autoimmune hypothyroidism [thyroid stimulating hormone (TSH) 74.64 uIU/mL (0.500-5.330 uIU/mL), thyroid peroxidase antibodies >900 IU/mL (<9 IU/mL)]. Her diabetes was treated with multiple daily subcutaneous injections of insulin aspart and insulin glargine (total daily dose approximately 1.0 units/kg/day), and she was taking levothyroxine 75 mcg/day for hypothyroidism. She presented to an outside hospital’s emergency department with nausea, vomiting, and hyperglycemia. She was found to be in severe DKA (pH 6.98, glucose 800 mg/dL, bicarbonate <5 mmol/L) and was treated with fluid resuscitation and an insulin infusion. She was transferred to our emergency department for admission to the Pediatric Intensive Care Unit. En route she suffered an apparent seizure with upper extremity tonic-clonic movements and dilated pupils that resolved spontaneously after approximately 20 seconds. She then had a decreased level of consciousness. Upon arrival to our emergency department, she exhibited altered mental status, tachycardia (heart rate 217 beats/minute), tachypnea (respiratory rate 42 breaths/minute), hypertension (blood pressure 150/70 mmHg), and was febrile to 40.1°C. Her initial laboratory evaluation showed an improvement in her metabolic acidosis (pH 7.06, bicarbonate 5 mmol/L) and hyperglycemia (glucose 339 mg/dL). A brain computed tomography (CT) scan was performed that showed no intracranial abnormalities. Thyroid studies were obtained that showed hyperthyroidism [fT4 2.9 ng/dL (0.6-1.4 ng/dL), total T3 210 ng/dL (80-170 ng/dL), TSH 0.163 uIU/mL (0.500-5.330 uIU/mL)]. She met the criteria for thyroid storm by scoring >45 points on the Burch-Wartofsky Point Scale [4] and satisfying the diagnostic criteria of the Japan Thyroid Association as she had thyrotoxicosis, symptoms involving the central nervous system, fever, tachycardia, and gastrointestinal symptoms [5]. She was continued on appropriate fluids and an insulin infusion for her DKA, and thyroid storm was treated with methimazole, potassium-iodide solution, methylprednisolone, and esmolol. She received an intravenous dose of levetiracetam for the concern of seizures. Her DKA resolved in <24 hours and she was transitioned to her home regimen of insulin. Her thyroid hormone levels became normal after 72 hours of therapy. A thyroid-stimulating immunoglobulin (TSI) index was normal at 21% of activity (normal <140%), and all antithyroid medications were discontinued. She denied that she had taken any additional doses of levothyroxine prior to this presentation. She was not restarted on levothyroxine at discharge.

The patient again presented in DKA but without thyroid storm two months and seven months after her episode of concurrent DKA and thyroid storm. Her thyroid hormone levels had remained in the normal range without supplemental levothyroxine during that time period.

The patient presented in DKA one month after her most recent presentation and was again in concurrent thyroid storm. Her initial laboratory evaluation showed pH 6.96, glucose 1,076 mg/dL, bicarbonate 7 mmol/L, fT4 4.6 ng/dL (0.6-1.4 ng/dL), total T3 270 ng/dL (80-170 ng/dL), TSH 0.275 uIU/mL (0.500-5.330 uIU/mL), and TSI index 16% (<140). She presented with abdominal pain, emesis, altered mental status (combative and agitated), questionable seizure activity, fever of 39.4°C, tachycardia (heart rate 198 beats/minute), and hypertension (blood pressure 150/80 mmHg). She again met the criteria for thyroid storm by the Burch-Wartofsky Point Scale and the Japan Thyroid Association. A brain CT scan was normal. She was treated with appropriate fluids, an insulin infusion, methimazole, hydrocortisone, and labetolol. Her DKA resolved in <24 hours and she resumed her usual home insulin regimen. Methimazole was continued until her fT4 and total T3 levels became low at 0.6 ng/dL and 71 ng/dL, respectively, after 10 days.

## Discussion

The disordered metabolic state of DKA is characterized by hyperglycemia, ketoacidosis, and ketonuria as a consequence of absolute or relative insulin deficiency accompanied by an increase in counterregulatory hormones (glucagon, cortisol, growth hormone, and epinephrine). This hormonal imbalance enhances hepatic gluconeogenesis, glycogenolysis, and lipolysis. An increase in intestinal glucose absorption, a decrease in insulin secretion and insulin half-life, and a decrease in the peripheral use of glucose due to insulin resistance are some of the several mechanisms by which hyperthyroidism can precipitate DKA in patients with diabetes. Thyroid hormones also increase hepatic glucose output and abnormal glucose metabolism via an increase in the hepatocyte plasma membrane concentrations of glucose transporter 2. Hepatic gluconeogenesis is further enhanced both by increased free fatty acids produced by increased catecholamine-stimulated lipolysis induced by the excess thyroid hormones, and by increased non-oxidative glucose disposal that results in an overproduction of lactate that enters the Cori cycle [3,6,7].

The exact pathophysiologic mechanism of thyroid storm is not clearly understood, but the transition from simple thyrotoxicosis to the crises of thyroid storm typically requires a stressful medical insult, such as uncontrolled hyperthyroidism, discontinuation of antithyroid drug, surgery, infection, trauma, or DKA [2]. Thyroid storm is characterized by dysfunction of multiple organs including the cardiovascular system, thermoregulatory system, gastrointestinal system, hepatic system, and central nervous system thought to be due to a drastic increase in the release of thyroid hormones, hyperactivity of the sympathetic nervous system, relative adrenal insufficiency, and increased peripheral cellular response to thyroid hormones [8]. Mortality rate is between 10% and 30% and is related to shock, multiple organ failure, or disseminated intravascular coagulation [9].

Interestingly, DKA can both trigger and delay or mask the diagnosis of thyroid storm in the setting of severe hyperglycemia [10]. Lower levels of free thyroid hormones have been associated with a higher degree of hyperglycemia and acidosis in children with DKA [11].

The diagnosis of thyroid storm is based on clinical findings and not solely on the severity of thyrotoxicosis. There is no level of thyroxine, triiodothyronine, or thyroid-stimulating hormone that indicates that this may be storm. The Burch-Wartofsky Point Scale (BPWS) helps to assess the probability of thyroid storm independently from the level of thyroid hormones by assigning a numerical value to specific clinical signs and symptoms. These criteria include hyperpyrexia, tachycardia, arrhythmias, congestive heart failure, agitation, delirium, psychosis, stupor, and coma, as well as nausea, vomiting, diarrhea, hepatic failure, and the presence of an identified precipitant. Points in the BWPS system are based on the severity of individual manifestations, with a point total of ≥45 consistent with thyroid storm, 25-44 points classified as impending thyroid storm, and <25 points making thyroid storm unlikely [3]. The Japanese Thyroid Association system uses combinations of similar clinical features to assign patients to the diagnostic categories of thyroid storm 1 (TS1) or thyroid storm 2 (TS2) [4]. Care should be taken with either system to avoid inappropriate application to patients without severe thyrotoxicosis because each of the manifestations of thyroid storm, with the possible exception of severe hyperpyrexia, may also be seen in the presence of any major illness, many of which are also known precipitants of thyroid storm [12].

Type 1 diabetes mellitus and autoimmune thyroid disease (AITD) in the same individual is considered as one variant of autoimmune polyglandular syndrome 3 (APS3), denominated as APS3 variant (APS3v) [13]. Hashimoto’s thyroiditis, estimated to occur in 17-30% of type 1 diabetes patients [14], and Graves’ disease, estimated to occur in approximately 0.5% of children with type 1 diabetes [15], are the most common types of AITD and are risk factors for development of thyroid storm. Graves’ disease is the most common etiology of hyperthyroidism escalating into thyroid storm [2].

The simultaneous development of DKA and thyroid storm is uncommon with 28 cases reported in the English-language literature compiled in two recent reviews, with the first case reported in 1957 [16,17]. Co-presentation of these two conditions almost always occurred in people with either preexisting diabetes, thyroid disease, or both. The majority of the reported patients had type 1 diabetes, all had Graves’ disease, and only two were in the pediatric age group at less than 18 years of age.

Our patient is unique from previous reported patients in several respects. She has had recurrent episodes of concurrent DKA and thyroid storm, which has thus far been unreported. One can only speculate regarding the cause of the patient’s recurrent thyrotoxic episodes.

All previous reported patients had Graves’ disease, but she apparently did not, as determined by TSI index levels being less than the baseline in bioassays. Her thyrotoxic states were short-lived that did not require antithyroid medication after resolution. She denied taking any additional levothyroxine prior to these episodes. Recurrent episodes of subacute or silent thyroiditis may have led to her transient thyrotoxic states; however, she did not have thyroid pain or tenderness nor did she have the hypothyroid phases that typically follow. Our patient was known to have considerable stress-related issues at home and at school, and there has been a case of psychological stress-induced Hashitoxicosis reported to occur 12 years after the initial diagnosis of autoimmune thyroiditis [18].

## Conclusions

Thyroid storm and DKA are both acute, potentially fatal complications of their respective preexisting conditions that share several presenting signs and symptoms. The coinciding development of both disorders is uncommon and may ultimately lead to significant morbidity and mortality. Early recognition and aggressive treatment of each entity is crucial to prevent further decompensation.
